# Canagliflozin Inhibits Human Endothelial Cell Inflammation through the Induction of Heme Oxygenase-1

**DOI:** 10.3390/ijms23158777

**Published:** 2022-08-07

**Authors:** Kelly J. Peyton, Ghazaleh Behnammanesh, Giovanna L. Durante, William Durante

**Affiliations:** Department of Medical Pharmacology and Physiology, University of Missouri, Columbia, MO 65212, USA

**Keywords:** canagliflozin, endothelial cells, heme oxygenase-1, bilirubin, inflammation, proliferation, migration

## Abstract

Sodium-glucose co-transporter 2 (SGLT2) inhibitors improve cardiovascular outcomes in patients with type 2 diabetes mellitus (T2DM). Studies have also shown that canagliflozin directly acts on endothelial cells (ECs). Since heme oxygenase-1 (HO-1) is an established modulator of EC function, we investigated if canagliflozin regulates the endothelial expression of HO-1, and if this enzyme influences the biological actions of canagliflozin in these cells. Treatment of human ECs with canagliflozin stimulated a concentration- and time-dependent increase in HO-1 that was associated with a significant increase in HO activity. Canagliflozin also evoked a concentration-dependent blockade of EC proliferation, DNA synthesis, and migration that was unaffected by inhibition of HO-1 activity and/or expression. Exposure of ECs to a diabetic environment increased the adhesion of monocytes to ECs, and this was attenuated by canagliflozin. Knockdown of HO-1 reduced the anti-inflammatory effect of canagliflozin which was restored by bilirubin but not carbon monoxide. In conclusion, this study identified canagliflozin as a novel inducer of HO-1 in human ECs. It also found that HO-1-derived bilirubin contributed to the anti-inflammatory action of canagliflozin, but not the anti-proliferative and antimigratory effects of the drug. The ability of canagliflozin to regulate HO-1 expression and EC function may contribute to the clinical profile of the drug.

## 1. Introduction

Diabetes mellitus is a major public health problem that affects an estimated 537 million people worldwide, and this number is projected to reach 643 million by 2030 [1]. It is responsible for 6.7 million deaths and caused at least 966 billion dollars in health care expenditures in 2021. Type 2 diabetes mellitus (T2DM) accounts for a vast majority of cases of hyperglycemia and the risk of getting this disease increases progressively throughout life. Cardiovascular disease is the leading cause of death in both men and women with diabetes mellitus [2]. Individuals with diabetes exhibit a two- to four-fold increase in mortality rate due to vascular complications, leading to a markedly attenuated life span [3,4,5]. Microvascular disorders, including retinopathy, nephropathy, and neuropathy, along with macrovascular complications such as ischemic heart disease, stroke, and limb ischemia account for much of the pathology associated with this metabolic disease [6,7]. While the mechanisms underlying vascular disease in diabetes are multifaceted, endothelial cell (EC) dysfunction is an early and pivotal event that contributes to the initiation and progression of diabetic vascular disease [8,9]. Several of the metabolic disturbances that occur in diabetes negatively impact EC function, however; hyperglycemia is believed to play a primary role [10]. Yet, strict glycemic control may not fully protect against the development of vascular disease, illuminating the need for additional therapeutic modalities to combat the vascular consequences of diabetes [11,12,13].

Sodium-glucose co-transporter 2 (SGLT2) inhibitors represent the latest class of glucose-lowering drugs that act by inhibiting glucose reabsorption in the proximal tubule of the kidney [14]. Intriguingly, several large cardiovascular outcome trials including the EMPA-REG-OUTCOME, CANVAS Program, and DECLARE-TIMI 58 found that SGLT2 inhibitors lower cardiovascular disease and/or mortality relative to other anti-glycemic agents in patients with T2DM [15,16,17]. More recently, a meta-analysis of these three clinical outcome trials revealed that SGLT2 inhibitors significantly reduced the incidence of myocardial infarction, stroke, and cardiovascular death in subjects with established atherosclerosis [18]. Moreover, ensuing observational studies have validated the beneficial effects of SGLT2 inhibitors on cardiovascular mortality, major adverse cardiovascular events, and hospitalization for heart failure [19,20].

Heme oxygenase-1 (HO-1) is a highly inducible enzyme that catabolizes heme to carbon monoxide (CO), ferrous iron, and biliverdin, which is reduced to bilirubin by biliverdin reductase [21]. HO-1 impedes the development of vascular disease by preventing apoptosis, inflammation, thrombosis, and oxidative stress in the vasculature [22,23,24,25]. In addition, HO-1 preserves blood flow at sites of arterial injury by limiting platelet aggregation and vascular cell growth and motility [26,27,28]. Interestingly, we recently identified canagliflozin as a novel inhibitor of vascular smooth muscle cell (SMC) proliferation and migration [29]. These actions are unique to canagliflozin as pharmacologically relevant concentrations of other SGLT2 inhibitors failed to modify SMC function. The study also found that canagliflozin uniquely stimulates the expression of HO-1 in SMCs via the transcriptional activation of the HO-1 gene via the reactive oxygen species (ROS)-Nrf2 signaling pathway and that the induction of HO-1 contributes to the anti-proliferative and anti-migratory actions of canagliflozin in these cells. These latter findings suggest that HO-1 may contribute to the clinical efficacy of canagliflozin in the circulation. However, it remains to be established whether canagliflozin regulates the expression of HO-1 in the vascular endothelium.

Several potential mechanisms have been proposed to explain the beneficial cardiovascular profile of SGLT2 inhibitors, including decreases in body weight, adipose tissue, plasma volume and lipids, blood pressure, renal disease, liver steatosis, increases in hematocrit, alterations in energy metabolism, and off-target effects of the drug [30,31,32,33]. However, the salutary effects of the drugs are independent of their glucose-lowering actions [34]. Emerging evidence also indicates that SGLT2 inhibitors affect EC function [33,35,36,37]. Indeed, studies from our laboratory and others found that the SGLT2 inhibitor canagliflozin blocks the proliferation and migration of ECs as well as endothelial inflammation in response to lipopolysaccharide and interleukin-1β [38,39,40]. Moreover, these actions on ECs are specific for canagliflozin and are not seen with other SGLT2 inhibitors, representing an off-target effect of the drug. While the ability of canagliflozin to suppress inflammation may contribute to its beneficial cardiovascular outcomes, our finding that canagliflozin also blocks EC proliferation and migration may explain the higher incidence of limb amputations observed with this drug [16,39]. The increased risk of limb amputation by canagliflozin has been attributed to reductions in blood limb flow in patients with T2DM that suffer from peripheral artery disease. By blocking EC growth and motility, key processes in angiogenesis, canagliflozin may further minimize limb blood flow in this patient population. Thus, the interaction between canagliflozin and ECs may dictate the cardiovascular actions of the drug. In the present study, we determined if canagliflozin stimulates the expression of HO-1 in human ECs. Furthermore, the role of HO-1 in mediating the biological actions of canagliflozin on ECs was investigated.

## 2. Results

**Figure 1:**
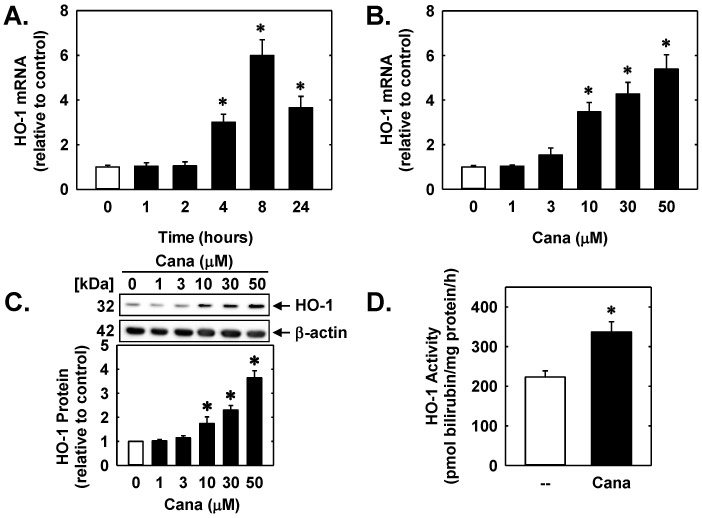
Canagliflozin (Cana) stimulates HO-1 expression in human ECs. (**A**) Cana (50 µM) stimulates a time-dependent increase in HO-1 mRNA expression. (**B**) Effect of Cana (0–50 µM) exposure for 8 h on HO-1 mRNA expression. (**C**) Effect of Cana (0–50 µM) exposure for 24 h on HO-1 protein expression. (**D**) Effect of Cana (50 µM) exposure for 24 h on HO activity. Results are mean ± SEM (*n* = 4–6). * Statistically significant effect of Cana.

Treatment of human ECs with canagliflozin stimulated a time-dependent increase in HO-1 mRNA (Figure 1A). A significant increase in HO-1 mRNA was first detected after 4 h of canagliflozin administration, peaked at 8 h, and transcript levels remained elevated following 24 h of drug treatment. Canagliflozin also stimulated a concentration-dependent increase in HO-1 mRNA (Figure 1B) and protein (Figure 1C) that resulted in a significant increase in HO activity (Figure 1D).

**Figure 2:**
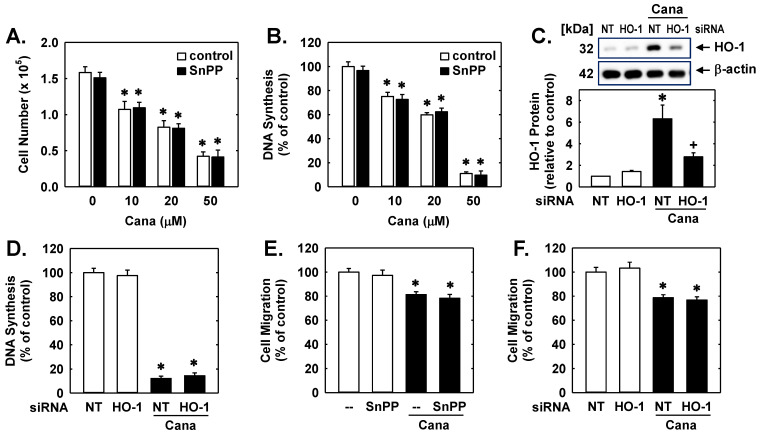
Canagliflozin (Cana) inhibits human EC proliferation, DNA synthesis and migration in a HO-1-independent manner. (**A**) Effect of Cana (0–50 µM) treatment for 96 h on cell proliferation in the presence and absence of the HO inhibitor SnPP (10 µM). (**B**) Effect of Cana (0–50 µM) treatment for 24 h on DNA synthesis in the presence and absence of SnPP (10 µM). (**C**) HO-1 expression in ECs transfected with NT or HO-1 siRNA (100 nM) and then exposed to CANA (50 µM) for 24 h. (**D**) Effect of HO-1 silencing on Cana-mediated inhibition of EC DNA synthesis. Cells were transfected with NT and HO-1 siRNA (100 nM) and then exposed to Cana (50 µM) for 24 h. (**E**) Effect of Cana (50 µM) treatment for 24 h on the migration of ECs in the presence and absence of SnPP (10 µM). (**F**) Effect of HO-1 silencing on Cana-mediated inhibition of EC migration. Cells were transfected with NT and HO-1 siRNA (100 nM) and then exposed to Cana (50 µM) for 24 h. Results are mean ± SEM (*n* = 4–6). * Statistically significant effect of Cana. ^+^ Statistically significant effect of HO-1 siRNA.

Since HO-1 is an established regulator of vascular cell growth and DNA synthesis [26,27,28], we determined whether the induction of HO-1 influences the capacity of canagliflozin to repress EC mitogenesis. Incubation of human ECs with canagliflozin resulted in a concentration-dependent inhibition of cell proliferation and DNA synthesis (Figure 2A,B). However, administration of the HO inhibitor SnPP had no effect on the basal rate of EC proliferation and DNA synthesis, nor did it affect the anti-proliferative action of canagliflozin. Transfection of ECs with HO-1 siRNA suppressed the canagliflozin-induced increase in HO-1 protein, confirming the efficacy of the knockdown approach (Figure 2C). Moreover, silencing HO-1 expression failed to modify baseline DNA production or the inhibition of DNA synthesis by canagliflozin (Figure 2D). Similarly, blocking HO-1 activity (Figure 2E) or knocking down HO-1 expression (Figure 2F) had no effect on the anti-migratory action of canagliflozin. In the absence of canagliflozin, SnPP or HO-1 siRNA minimally affected EC migration.

**Figure 3:**
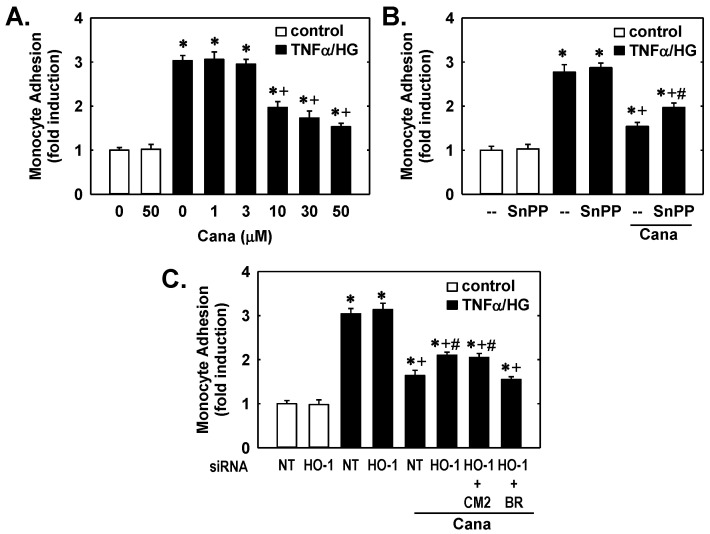
HO-1 contributes to the anti-inflammatory action of canagliflozin (Cana). (**A**) Effect of Cana (0–50 µM) on monocyte adhesion following treatment of ECs with TNFα (10 ng/mL) and a high concentration of glucose (HG; 25 mM) for 24 h. (**B**) Effect of HO-1 inhibition on Cana-mediated blockade of monocyte adhesion. Cells were exposed to TNFα (10 µM)/HG (25 mM) in the absence and presence of Cana (50 µM) and/or SnPP (10 µM) for 24 h. (**C**) Effect of HO-1 silencing on Cana-mediated inhibition of monocyte adhesion. Cells were transfected with NT or HO-1 siRNA (100 nM) and then exposed to TNFα (10 µM)/HG (25 mM) in the absence and presence of Cana (50 µM), CORM2 (CM2; 20 µM), and/or bilirubin (20 µM) for 24 h. Results are mean ± SEM (*n* = 6). * Statistically significant effect of TNFα/HG. ^+^ Statistically significant effect of Cana. ^#^ Statistically significant effect of SnPP or HO-1 siRNA.

Finally, we investigated the capacity of canagliflozin to block inflammatory responses in ECs. Cells were treated with TNFα and a high concentration of glucose to mimic the environment encountered by ECs in patients with T2DM. Incubation of ECs with this cytokine/high glucose cocktail resulted in a striking increase in monocyte adhesion that was blocked by canagliflozin in a concentration-dependent fashion (Figure 3A). However, canagliflozin had no effect on monocyte adhesion in the absence of the cytokine/high glucose mixture. Interestingly, HO-1 inhibition or knockdown attenuated the anti-inflammatory effect of canagliflozin but failed to modulate monocyte adhesion in the absence of canagliflozin (Figure 3B,C). Moreover, the addition of bilirubin, but not CORM2, reversed the increase in monocyte adhesion in HO-1-silenced ECs treated with canagliflozin.

## 3. Discussion

The present study identifies canagliflozin as a novel inducer of HO-1 in human ECs. It also found that that the induction of HO-1 contributes to the anti-inflammatory action of canagliflozin in ECs via the generation of bilirubin. In contrast, HO-1 does not mediate the anti-proliferative and anti-migratory effects of canagliflozin on the vascular endothelium. Thus, the ability of canagliflozin to evoke anti-inflammatory effects via HO-1 may contribute to its beneficial cardiovascular profile, whereas the negative, HO-1-independent effects of canagliflozin on EC growth and motility may promote an elevated risk for limb amputation in diabetes [16]. Collectively, this work illustrates the potential utility of pharmacologically targeting HO-1 in ameliorating diabetes-associated vascular inflammation. It also highlights novel interactions between canagliflozin and HO-1 in human ECs.

Although canagliflozin was designed to target SGLT2, a growing list of off-target proteins have been identified [29,32]. In the current study, we show that canagliflozin is capable of stimulating HO-1 expression in human ECs. The induction of HO-1 occurs in a concentration and time-dependent manner and results in a significant rise in cellular HO activity. Notably, the induction of HO-1 is observed with a clinically relevant concentration of canagliflozin (10 µM) [41]. The ability of canagliflozin to elevate HO activity may contribute to the rise in circulating bilirubin detected in diabetic patients prescribed the drug [42,43]. While the molecular mechanism that underlies the stimulation of endothelial HO-1 expression was not determined, it likely involves the activation of Nrf2 by ROS, since we recently determined that this signaling pathway is responsible for mediating the induction of HO-1 in canagliflozin-treated vascular SMCs [29]. Consistent with our findings in vascular cells, canagliflozin promotes the nuclear translocation of Nrf2 and the expression of HO-1 in cardiac cells [44], suggesting that the induction of HO-1 is a uniform response to this drug in cardiovascular tissues.

We also found that a clinically relevant concentration of canagliflozin attenuates monocyte adhesion onto ECs that were exposed to a diabetic milieu. This extends earlier work demonstrating that canagliflozin protects cultured ECs against inflammatory stress by lipopolysaccharide and interleukin-1β [38,40]. Significantly, the induction of HO-1 in ECs contributes to the anti-inflammatory effect of canagliflozin, as inhibition of HO-1 expression represses the ability of canagliflozin to block monocyte adhesion to ECs treated with TNFα and a high concentration of glucose. However, exogenous administration of bilirubin, but not CO, can substitute for HO-1 and restore the anti-inflammatory effect of canagliflozin, suggesting that HO-1-derived bilirubin is responsible for the anti-inflammatory action of canagliflozin. This is consistent with numerous reports illustrating the anti-inflammatory properties of this bile pigment in the vascular endothelium [45,46,47,48]. Notably, silencing HO-1 expression does not fully eliminate the anti-inflammatory action of canagliflozin, suggesting that other factors may also support the drug’s ability to avert inflammation. In this respect, recent reports have implicated a role for AMP-activated protein kinase (AMPK), hexokinase II, and sirtuin-6 in conveying the anti-inflammatory action of canagliflozin in ECs [38,40,49]. In addition, the AMPK-Akt-endothelial nitric oxide synthase signaling pathway has been shown to underlie the anti-inflammatory action of canagliflozin in both the heart and kidney [44,50]. Canagliflozin also exerts an anti-inflammatory effect by inhibiting intracellular glucose metabolism and promoting autophagy in immune cells by modulating alveolar macrophage polarization towards the M2 phenotype, and by blocking the assembly of the inflammasome [51,52,53]. Thus, canagliflozin alleviates inflammation in T2DM via multiple mechanisms.

The induction of HO-1 in ECs elicits many beneficial effects in the circulation. Aside from limiting inflammation, the upregulation of HO-1 by canagliflozin attenuates oxidative stress in diabetes leading to increases in nitric oxide bioavailability and improvements in endothelium-dependent vasodilation and hypertension [22,23,24,25]. In addition, the rise in endothelial HO-1 exerts an anti-thrombogenic effect by blocking platelet activation and aggregation and preventing oxidative damage to the endothelium [54]. Moreover, endothelial HO-1 activity restrains SMC proliferation and migration and arterial lesion formation through the release of CO and bilirubin [26,27,28,55,56]. The release of bilirubin by ECs may also curb the accelerated rate of atherosclerosis in diabetes through its impact on adhesion molecule expression as well as cholesterol metabolism [57,58]. Intriguingly, bilirubin improves renal blood flow, renal vascular resistance, and glomerular filtration rate, and this may underlie the reduced risk for kidney failure in patients with T2DM and nephropathy receiving canagliflozin [59,60,61,62]. Recently, bilirubin was demonstrated to be a potent activator of peroxisome-proliferator-activated receptor-α (PPARα), triggering the transcription of a host of PPARα target genes that regulate whole-body energy homeostasis, including fibroblast growth factor 21 [63]. Bilirubin treatment decreases fasting glucose, body weight, body fat and increases fatty acid oxidation in mice: effects that are mirrored by canagliflozin treatment [16,62,64] Collectively, these actions of HO-1 and its products may contribute to the beneficial cardiometabolic outcomes in patients with T2DM that are administered canagliflozin.

The study also reveals that HO-1 does not contribute to the anti-proliferative and anti-migratory action of canagliflozin in ECs. This is in contrast to our work demonstrating a role for HO-1 in mediating the inhibitory effects of canagliflozin on the proliferation and migration of vascular SMCs [29]. This discrepancy may reflect the disparate effects of HO-1 on the growth and migration of vascular cells [27]. While HO-1 is a well-established inhibitor of SMC proliferation and migration, most, but not all, studies indicate that HO-1 and its reaction products stimulate EC growth and motility [26,27,28,65,66,67,68,69,70]. Failure of HO-1 to modify the proliferative and migratory action of canagliflozin in ECs may reflect the variable regulatory effect of the enzyme in these cells. Alternatively, the levels of HO-1 may be insufficient to modulate EC growth and locomotion, and/or canagliflozin may mobilize other more potent pathways that govern these EC functions. In this regard, the activation of AMPK by canagliflozin may be significant, as we previously identified this kinase as a potent inhibitor of EC proliferation and migration [71]. Furthermore, the capacity of canagliflozin to block the metabolism of glutamine may be relevant as the flux of this amino acid through the tricarboxylic acid cycle plays an essential role in promoting EC proliferation and migration [72,73]. The finding that canagliflozin impairs EC proliferation and migration is of potential concern, as it may compromise the reendothelialization of arteries following coronary stenting. Moreover, it may exacerbate peripheral artery disease in diabetes by constraining angiogenesis, leading to increases in the risk of limb amputations [16,39].

In conclusion, the present study identifies canagliflozin as a pharmacologically relevant inducer of HO-1 in the human vascular endothelium. It also found that HO-1-derived bilirubin contributes to the anti-inflammatory action of canagliflozin, but the enzyme does not play a role in transducing the anti-proliferative and anti-migratory actions of the drug. The ability of canagliflozin to regulate HO-1 expression and EC function may contribute to the clinical effects of the drug in patients with T2DM.

## 4. Materials and Methods

### 4.1. Reagents

M199 medium, gelatin, penicillin, streptomycin, heparin, NaOH, sodium dodecylsulfate (SDS), Tris, phosphate-buffered saline (PBS), ethylendiaminetetraacetic acid (EDTA), chloroform, glycerol, trichloroacetic acid, trypsin, hydroxyurea, glucose, bromophenol blue, lipofectamine, CO-releasing molecule-2 (CORM2), NADPH, glucose-6-phosphate, glucose-6-phosphate dehydrogenase, Triton X-100, and mercaptoethanol were from Sigma-Aldrich (St. Louis, MO, USA). Bilirubin and tin protoporphyrin were from Frontier Scientific (Logan, UT, USA). The antibody against HO-1 was from Assay Designs (Ann Arbor, MI, USA) and the antibody against β-actin was from Santa Cruz (Santa Cruz, CA, USA). Tumor necrosis factor-α (TNFα) was from R&D Systems (Minneapolis, MN, USA). Canagliflozin was purchased from Selleck Chemicals (Houston, TX, USA). [^3^H]Thymidine was from Perkin Elmer (Boston, MA, USA).

### 4.2. Cell Culture

Human umbilical vein ECs were purchased from Lonza Incorporated (Allendale, NY, USA) and were cultured on gelatin-coated plates in M199 medium supplemented with 20% bovine calf, 2 mM L-glutamine, 50 µg/mL EC growth factor, 90 µg/mL heparin and 100 U/mL penicillin and streptomycin (GLS1 paper). The human monocytic cell line U937 (American Type Culture Collection, Manassas, VA, USA) was grown in suspension in RPMI-1640 medium containing 2 mM L-glutamine, 1 mM sodium pyruvate, 4.5 g/L glucose, 10% fetal bovine serum, and 100 µM penicillin and streptomycin. All cells were grown in an atmosphere of 95% air and 5% CO_2_ at 37 °C [74].

### 4.3. Cell Proliferation and DNA Synthesis

ECs were seeded (2 × 10^4^ cells/well) onto six-well plates in serum containing media and grown overnight. Cells were then washed and treated with various reagents. Media with appropriate additions were replenished every second day. After four days, cell number measurements were made by dissociating cells with trypsin (0.05%): EDTA (0.53 mM) and counting cells using an automated cell counter (Moxi Z ORFLO Technologies, Ketchum, ID, USA). In addition, DNA synthesis was monitored by pulsing ECs with [^3^H]thymidine (1 µCi/mL), as we previously described [72].

### 4.4. Cell Migration

EC migration was determined using a scratch-wound assay [72]. Confluent cell monolayers were scratched with a sterile pipet tip to generate a wound approximately 0.1 cm in width. The medium was discarded, and cell debris removed by several washes with PBS. Injured monolayers were treated with hydroxyurea (0.5 mM) to prevent cell growth. Phase-contrast images of the injured area were obtained immediately and 24 h following injury with an inverted microscope equipped with a digital camera (Q-Imaging, QICAM; Hitschfel Instruments Incorporated, St. Louis, MO, USA). The degree of wound closure was determined by planimetry.

### 4.5. Monocyte Adhesion

U937 cells were labeled with [^3^H]thymidine (1 µCi/mL for 24 h) and layered onto confluent EC monolayers that had been treated with canagliflozin [75]. The monocytes were allowed to adhere for one hour at 37 °C in the presence and absence of CORM2 or bilirubin. Non-adherent U937 cells were removed by washing cells three times with PBS and adherent monocytes quantified by liquid scintillation counting (Tricarb liquid scintillation analyzer, model 2100, Packard, Meriden, CT, USA).

### 4.6. Western Blotting

ECs were collected in lysis buffer (125 mM Tris [pH 6.8], 12.5% glycerol, 2% SDS, and bromophenol blue, boiled for 5 min and proteins resolved by SDS-polyacrylamide gel electrophoresis. Following transfer, nitrocellulose membranes were blocked with PBS containing Triton X-100 (0.25%) and non-fat milk (5%) for one hour at room temperature and incubated overnight at 4°C with antibodies against HO-1 (1:1500) or β-actin (1:1000). Membranes were then washed, incubated with horseradish peroxidase-conjugated secondary antibodies (1:2000) and immunoreactive bands detected using commercial chemoluminescence reagents (Amersham, Arlington Height, IL, USA). Protein expression was quantified by densitometry and normalized with respect to β-actin.

### 4.7. Quantitative Real-Time PCR

Total RNA was extracted from ECs with TRIzol reagent and reverse transcribed to cDNA using a transcription kit and random hexamer primers (Thermo Fisher Scientific, Waltham, MA, USA). Real-time PCR was conducted with a SYBR Green Supermix in a SYBER Green Cycler iQ 5RT-PCR detection system (Bio-Rad Laboratories, Hercules, CA, USA). The primer sequences were: HO-1, forward GTACTTTGGTGCCTACTCCA and reverse CGGCCCGAACATAGTAATTC and 18S RNA, forward TCAAGAACGAAAGTCGGAGG and reverse GGACATCTAAGGGCATCACA. Transcript levels were quantified using the 2^−∆∆CT^ method and normalized to 18S RNA, as we previously described [74].

### 4.8. HO Activity

HO activity was determined by absorption spectroscopy, as we have previously outlined [76]. ECs were harvested and sonicated in a phosphate (100 mM) buffer (pH 7.4) containing MgCl_2_ (2 mM), NADPH (0.8 mM), glucose-6-phosphate (2 mM), glucose-6-phophate dehydrogenase (0.2 U), and rat liver cytosol (2 mg) as a source of biliverdin reductase, and the substrate heme (20 µM). The reaction proceeded for one hour at 37 °C in the dark and was terminated by the addition of chloroform. The extracted bilirubin was measured by the difference in absorption between 464 and 530 nm with an extinction coefficient of 40 mM^−1^ cm^−1^. The protein concentration in each sample was determined with a Bradford protein assay and HO activity expressed in picomoles of bilirubin formed per milligram of protein per hour.

### 4.9. Gene Silencing

Gene expression was silenced using siRNA targeting HO-1. Non-targeting and HO-1 siRNA were obtained from Dharmacon (Lafayette, CO, USA) and transfected into ECs (100 nM), using lipofectectamine, as we previously reported [76].

### 4.10. Statistical Analysis

Data are presented as mean ± SEM. Statistical analyses were performed with the use of a Student’s *t*-test and one way analysis of variance with the Holm-Sidak post-hoc test when more than two treatments were compared. The results were considered significant when *p* values were less than 0.05.

## Data Availability

All the data is contained within the article.
